# Suppression of mRNA Nanoparticle Transfection in Human Fibroblasts by Selected Interferon Inhibiting Small Molecule Compounds

**DOI:** 10.3390/biom7030056

**Published:** 2017-07-31

**Authors:** Yang Liu, Manoj N. Krishnan, Kyle K. L. Phua

**Affiliations:** 1 Department of Chemical and Biomolecular Engineering, Faculty of Engineering, National University of Singapore, 1 Engineering Drive 3, Singapore 117580, Singapore; johnliuyang@nus.edu.sg; 2 Program on Emerging Infectious Diseases, Duke-NUS Medical School, 8 College Road, Singapore 169857, Singapore; manoj.krishnan@duke-nus.edu.sg

**Keywords:** small molecules, mRNA delivery, interferon, transfection

## Abstract

In vitro transcribed (IVT) mRNA is increasingly applied in lieu of DNA to deliver reprogramming genes to fibroblasts for stem cell derivation. However, IVT mRNA induces interferon (IFN) responses from mammalian cells that reduces transfection efficiency. It has been previously suggested that small molecule inhibitors of IFN are a viable strategy to enhance mRNA transfection efficiency. Herein, we screen a list of commercially available small molecules, including published IFN inhibitors, for their potential to enhance mRNA transfection in BJ fibroblasts. Transfection enhancement is quantified by relative mean fluorescence intensity of translated green fluorescent protein (GFP) in treated cells compared to dimethyl sulfoxide treated controls. Within toxicological constrains, all tested small molecules did not enhance mRNA transfection in BJ fibroblasts while a third of the tested compounds unexpectedly inhibited GFP expression even though IFN-β production is inhibited. Based on the results of our study, we conclude that small molecule inhibitors, including IFN inhibitors, tested in this study do not enhance in vitro mRNA transfection efficiency in human fibroblasts.

## 1. Introduction

The field of mRNA therapeutics has gained significant interest from both academia and industry in recent years as dozens of clinical trials and biotech companies have been created to evaluate the clinical efficacy of mRNA as a therapeutic [1]. In vitro transcribed (IVT) mRNA holds two key advantages in applications that require transient exogenous gene expression: higher transfection efficiency in non-dividing cells and higher safety profile compared to plasmid DNA [2]. To date, many biomaterials-based strategies have been developed to overcome mRNA delivery barriers such as uptake and endosome escape [3,4,5,6,7,8]. However, these strategies fail to address mRNA induced anti-viral response, which is a major barrier unique to therapeutic application of mRNA [9]. mRNA induces anti-viral responses in transfected cells through the activation of pathogen associated molecular patterns (PAMPs), such as toll-like receptors (TLRs) and RIG-I-like receptors (RLR), resulting in the production of interferon (IFN). IFN induces an antiviral state in transfected cells (through upregulation of protein kinase RNA activated, PKR), which leads to reduced mRNA translation into proteins [10,11].

Currently, one of the most promising and widely adopted strategies to overcome suppression of mRNA translation by IFN is the substitution of uridine triphosphate and cytidine triphosphate with naturally occurring pseudouridine (ψ) triphosphate and/or 5-methyl-cytidine triphosphate (m5C) [11] , respectively, during in vitro transcription. The resultant modified mRNA significantly reduces IFN production that normally occurs with unmodified mRNA, leading to diminished PKR activation and higher mRNA transfection efficiency [11]. The mechanism of action is attributed to passive evasion of PAMPs, which stimulate IFN production through various IFN activation pathways. In this report, we hypothesize that active inhibition of PAMPs and/or the IFN activation pathway is also a reasonable strategy to enhance mRNA transfection. Indeed, this concept of active inhibition was recently reviewed [9] and a proof-of-principle study focused on the use of small molecules Bay11 and BX795 were reported [12]. Interestingly, many other small molecules that had been reported to inhibit IFN were also available commercially. We hypothesize that some of these compounds could potentially be developed into therapeutic agents to enhance mRNA transfection efficiency without inducing excessive toxicity.

In this report, we screened a list of small molecules that interfere with PAMPs and/or IFN activation for their potential application in enhancing mRNA transfection. We first performed a toxicity screen of these small molecules on BJ fibroblasts to ascertain safe treatment durations and working concentrations. Using green fluorescent protein (GFP) mRNA as a reporter gene, transfection efficiency was quantified via flow cytometry based on mean fluorescence intensity in transfected cells. IFN-β production during transfection was quantified using enzyme-linked immunosorbent assay (ELISA).

## 2. Results

### 2.1. Cytotoxicity Profiles of Small Molecule Compounds

We first examined cytotoxicity induced by small molecules on BJ fibroblasts to determine suitable treatment concentration and duration. Two durations were chosen: 4 h, which corresponded to the incubation time with mRNA nanoparticles in a typical transfection experiment and 16 h, which corresponded to overnight incubation. BJ fibroblasts were treated with small molecule compounds for both durations followed by cell viability assay. Cells were further incubated in complete media without small molecules for another 48 h followed by a second cell viability assay.

**Figure 1:**
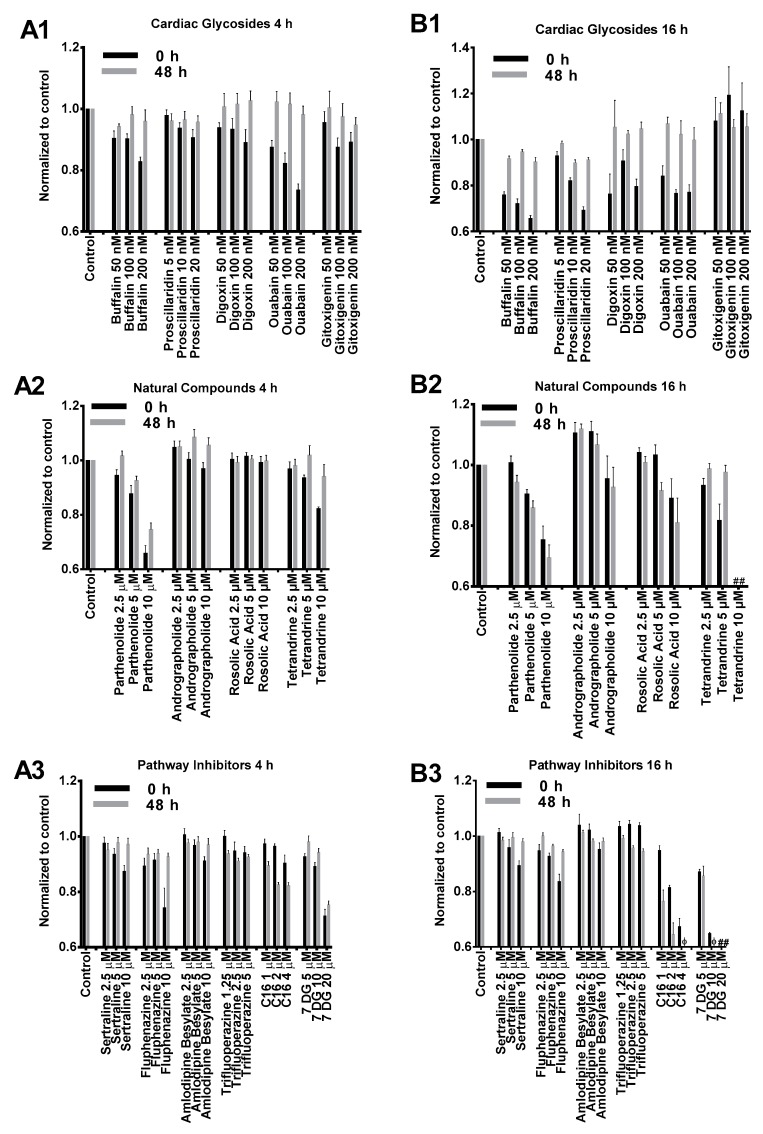
Cytotoxicity profiles of small molecules listed in Table 1. BJ cells were treated with small molecules or dimethyl sulfoxide (DMSO) control for 4 h (**A1–A3**) or 16 h (**B1–B3**) followed by cell viability assay (0 h, black columns). Cells were further incubated in complete media without small molecules for another 48 h (grey columns) followed by a second cell viability assay. Cell viability results were normalized to respective DMSO controls in each experiment. # and φ indicate relative viability of <10% and <50% compared to DMSO controls, respectively.

As illustrated in Figure 1, the cytotoxicity profiles of most compounds tested in our study presented similar dose-dependent cytotoxicity. BJ cells treated with cardiac glycosides initially showed reduced viability but significantly recovered after 48 h incubation (Figure 1(A1,B1)). This indicated that the effects of cardiac glycosides on cellular metabolism were reversible. Except for gitoxigenin, all other cardiac glycosides severely impacted cell metabolism after 16 h treatment as cell viability dropped below 80% compared to control. However, such effects were temporary because cell viability measurements substantially recovered afterwards (Figure 1(B1)). The effects of natural compounds on BJ cells were similar at low to medium concentrations for both 4 h and 16 h treatment durations (Figure 1(A2,B2)). However, cells treated for 16 h at high concentrations presented notable toxicity. In particular, acute toxicity was observed (<10% viability compared to control) when cells were treated with tetrandrine at 10 μM for 16 h. PKR inhibitor imidazolo-oxindole C16 and 7-Desacetoxy-6,7-dehydrogedunin (7DG) were generally well tolerated at low to medium concentrations for 4 h (Figure 1(A3)). However, significant cytotoxicity was observed after 16 h treatment and unlike cardiac glycosides, no recovery was observed after 48 h incubation (Figure 1(B3)). On the other hand, TLR3 inhibitors (sertraline, fluphenazine, amlodipine besylate and trifluoroperazine) applied at their respective concentrations did not present significant toxicity for both treatment durations (Figure 1(A3,B3)). Based on these cytotoxicity profiles, we chose to apply 4 h treatment duration with medium concentrations for transfection studies as these were the most tolerable conditions for all tested small molecules. 

**Figure 2:**
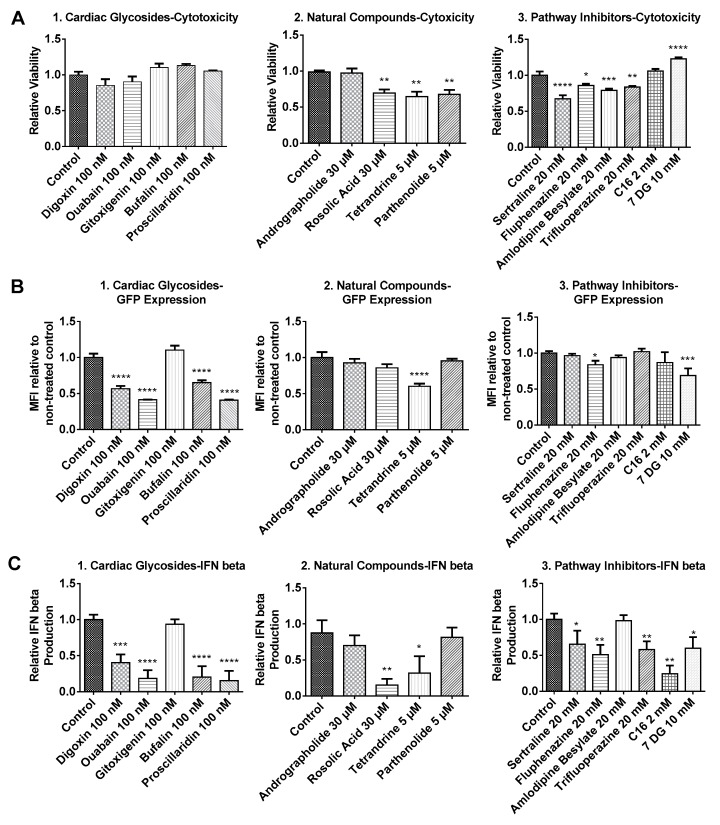
Relative cell viability (**A**), relative green fluorescent protein (GFP) expression (**B**) and relative interferon (IFN)-β production (**C**) of BJ fibroblasts transfected with GFP mRNA following small molecules treatment. Cells were incubated with small molecules for 1h followed by a 3 h transfection with GFP mRNA nanoparticles in the presence of the small molecules. Supernatants were then collected for IFN-β measurement via enzyme-linked immunosorbent assay (ELISA). Cells were further incubated in complete media without small molecules for another 4 h before being assayed for cell viability and GFP expression, respectively. Results were normalized to the average values of control groups that were transfected without small molecules treatment. Histograms of relative GFP expression can be found in Figure S1. * *p* < 0.05; ** *p* < 0.01; *** *p* < 0.005; **** *p* < 0.0001. MFI: Mean Fluorescence Intensity.

Considering that mRNA transfection together with small molecules treatment might result in additional cytotoxicity in BJ fibroblasts, cell viability was re-examined before transfection efficiency was assayed via flow cytometry. As shown in Figure 2(A1), cardiac glycosides applied together with mRNA nanoparticles did not affect cell viability. For andrographolide, rosolic acid and TLR3 inhibitors (sertraline, fluphenazine, amlodipine besylate and trifluoroperazine), we found that medium concentrations of these six small molecules identified in Figure 1 were too low because they failed to elicit significant changes in both transfection efficiency and IFN production. In order to elicit an effect (in either transfection efficiency, IFN production or cell viability), dosages of these six small molecules were increased to concentrations indicated in Figure 2(A2, A3) after further literature review [13,14,15]. For natural compounds, it was observed that tetrandrine, parthenolide and rosolic acid induced some toxicity. Nevertheless, cell viability remained above 60% compared to control. On the other hand, andrographolide remains non-toxic to cells even when its concentration was tripled from 10 μM to 30 μM. For pathway inhibitors, except for C16 and 7DG, all TLR inhibitors induced toxicity although average cell viability also remained above 60% compared to control. Interestingly, cells treated with 7DG exhibited promoted cellular viability, which might be attributed to the inhibition of caspase 1 activation [16,17].

### 2.2. BJ Fibroblasts Transfected in the Presence of Small Molecules Express Less GFP Even with Reduced IFN Production

**Figure 3:**
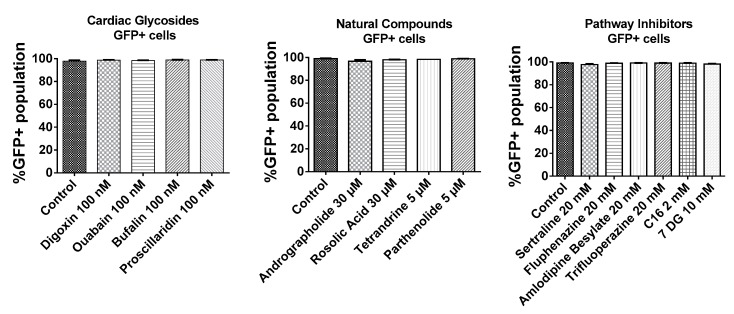
Transfection efficiency of BJ fibroblasts based on %GFP+ population. BJ fibroblasts were incubated with small molecules for 1h followed by a 3 h transfection with GFP mRNA nanoparticles in the presence of the small molecules. Cells were further incubated in complete media without small molecules for another 4 h and analyzed by flow cytometry.

As shown in Figure 3, BJ fibroblasts were very efficiently transfected as >95% of cells expressed GFP. In addition, small molecules tested in this study did not interfere with the number of GFP transfected cells. Given that almost every cell was transfected with GFP mRNA, the mean fluorescence intensities of the total live cell population were compared to ascertain effects of small molecules on the expression of GFP mRNA.

As shown in Figure 2B, we determined that small molecules applied in this study did not promote, while some even inhibited the expression of GFP mRNA. Most cardiac glycosides inhibited GFP mRNA expression significantly. The only exception was gitoxigenin, where no enhancement or inhibition effects were observed. Nevertheless, a modest reduction in GFP expression was observed when up to 10 μM of gitoxigenin was applied (data not shown), indicating that effects of gitoxigenin remained consistent with other cardiac glycosides. For natural compounds, even at higher concentrations, no enhancement in GFP expression was observed. Notably, rosolic acid and tetrandrine reduced IFN production but this correlated with either no change or reduced GFP expression (Figure 2(B2, C2)). For TLR3 inhibitors (sertraline, fluphenazine, amlodipine besylate and trifluoroperazine), when higher concentrations were applied, there was statistically significant inhibition of IFN production in all except amlodipine besylate (Figure 2(C3)). However, no enhancement in GFP expression was observed in all except fluphenazine, where there was a slight reduction in GFP expression (Figure 2(B3)). For PKR inhibitors (C16 and 7DG), C16 had no effect while 7DG inhibited GFP expression (Figure 2(C2)). Nevertheless, both inhibited IFN production efficiently (Figure 2(C3)).

## 3. Discussion

Although IVT mRNA has emerged as a promising tool for non-viral gene delivery, anti-viral responses triggered by mRNA compromise the translation of the desired protein and pose as a delivery barrier for non-vaccine applications [9]. This is particularly problematic for serial transfections needed in applications such as cellular reprogramming. The idea of enhancing mRNA transfection with IFN inhibiting small molecules is compelling as it is a cheap and scalable method to reduce IFN production in mRNA transfected cells, leading to an expected improvement in mRNA transfection efficiency through reduction of PKR activation [18]. It is also an orthogonal approach which may be applied together with established strategies such as modified mRNA and B18R (a secreted protein that binds to extracellular IFN molecules and blocks them from IFN receptors) for potential synergistic effects. Moreover, one-third of the small molecules selected in this study are approved by the Food and Drug Administration (FDA) (Table 1). Hence, if found effective, they can be more easily translated to the clinics for mRNA-based gene therapy applications via drug repurposing. Despite these advantages, there has not been a study that directly and systematically correlates transfection efficiency with IFN inhibiting small molecules. Interestingly, a few of these compounds have been reported to inhibit the production of type I IFN albeit in different cell types, but it remains to be determined whether they will be equally effective on human fibroblasts.

In this study, we applied a direct approach to correlate the effects of small molecule IFN inhibitors on mRNA transfection in BJ fibroblasts. Small molecules are grouped into three categories: cardiac glycosides (with differing chemical structures based on the size of the lactone ring and presence of conjugated sugar residues), natural compounds and pathway (TLR3 and PKR) specific inhibitors. The cardiac glycosides are published IFN inhibitors [20] while others are inhibitors of the dsRNA or IFN activated pathways [15,17,22,23,24,25,26,27]. Their effects are evaluated based on GFP expression, IFN production, and cell viability. An ideal small molecule inhibitor will inhibit IFN production while increasing GFP expression without significantly affecting cell viability. 

However, none of the small molecules that we have tested are able to enhance in vitro mRNA transfection efficiency on BJ fibroblasts. Cardiac glycosides, rosolic acid, tetrandrine, PKR inhibitors and some TLR3 inhibitors are capable of inhibiting IFN production in BJ fibroblasts. However, they either have no effect (rosolic acid, sertraline, trifluoperazine and C16) or unexpectedly reduce GFP expression (cardiac glycosides, tetrandrine, fluphenazine and 7DG). Parthenolide and amlodipine besylate are ineffective on BJ fibroblasts as no enhancement in GFP expression can be achieved (Figure 2B) even at toxic concentrations (Figure 2A).

## 4. Materials and Methods

### 4.1. Materials

Ouabain octahydrate, bufalin, digoxin and fluphenazine dihydrochloride were purchased from MP Biomedicals (Singapore). Gitoxigenin, proscillaridin, andrographolide, *p*-Rosolic acid, tetrandrine, 7DG, sertraline hydrochloride and amlodipine besylate were purchased from Sigma-Aldrich (Singapore). PKR inhibitor (C16) was purchased from Merck (Singapore). Parthenolide and trifluoperazine hydrochloride were purchased from Caymen Chemicals (Ann Arbor, MI, USA). DMSO (Sigma-Aldrich) was used to dissolve small molecules to establish stock solutions. Resazurin stock solution was prepared by dissolving 1 g of resazurin sodium salt (MP Biomedicals) in 100 mL sterile phosphate buffered saline (PBS) and filtered through a 0.22 µm filter. Resazurin working solution (cell viability assay reagent) was freshly prepared prior to each experiment by diluting stock solution with growth medium at a ratio of 1:250. Cell viability assay reagent applied at this ratio was tested on BJ fibroblasts and determined to vary linearly with cell number. Dulbecco’s Modified Eagle’s medium (DMEM) with high glucose, penicillin-streptomycin 100× solution, fetal bovine serum (FBS) and trypsin 2.5% 10× solution were purchased from Hyclone (GE Healthcare Life Sciences, Marlborough, MA, USA). 

### 4.2. GFP mRNA Synthesis

Cloning of GFP transcription template and synthesis of GFP mRNA were performed as previously described [28,29]. Briefly, a GFP plasmid containing a T7 promoter and polyA tail (64 residues) was linearized with *Spe*I (New England Biolabs, Singapore). After purification, DNA was used as template for in vitro transcription using T7 High Yield RNA Synthesis Kit in the presence of anti-reverse cap analogue (ARCA) according to the manufacturer’s protocol with a capping efficiency of ~80% (based on 3:1 ratio of ARCA cap to GTP). IVT mRNA was purified with RNEasy kit (Qiagen, Singapore), quantified by spectrophotometry and analyzed by agarose gel electrophoresis to confirm the synthesis of full-length mRNA.

### 4.3. Cell Culture and GFP mRNA Transfection

BJ fibroblasts were purchased from the American Type Culture Center (ATCC, Singapore) and cultured in DMEM growth medium supplemented with 10% heat-inactivated FBS, 100 units/mL penicillin and 100 μg/mL streptomycin at 37 °C in a saturated humidified atmosphere with 5% CO_2_. BJ fibroblasts were seeded on 24-well plates with a cell density of 6 × 10^4^ cells/well. Cells were then treated with small molecules one hour before GFP mRNA transfection (250 ng/well) using Stemfect Transfection Kit (Stemgent, Lexington, MA, USA). Supernatants were collected 3 h after transfection to determine IFN production using ELISA. Cells were incubated in fresh medium for another 4 h prior to cell viability assay, followed by cell harvesting and fixation at the end of experiments. GFP expression was then analyzed by flow cytometry. 

### 4.4. Cytotoxicity Assay

Cells were seeded on 96-well plates at a density of 1 × 10^4^ cells/well in a final volume of 100 µL and incubated overnight. Growth medium was replaced by fresh medium containing either a compound from Table 1 or negative control (DMSO) with 4 replicates (*n* = 4) in each treatment group. Cells were then incubated for another 4 or 16 h before further analysis. The final concentration of DMSO was identical in each well. Cytotoxicity assay was performed at the end of small molecule treatment (0 h) as well as after 48 h in growth medium. At each indicated time point, medium was removed completely and cells were washed with PBS before adding 100 µL of resazurin working solution into each well. After incubating with resazurin for 2 h, fluorescence signals were measured using 544 nm excitation and 590 nm emission filter settings on a FLUOstar OPTIMA spectrophotometer (BMG LABTECH, Ortenberg, Germany). Three blank wells without cells were used for subsequent subtraction of background. 

### 4.5. Production of IFN-β

IFN-β production in the supernatant collected 3 h after transfection was detected via standard sandwich ELISA protocol using a capture antibody, rabbit polyclonal to interferon-β (C-terminal; ab186669) and a detection antibody, rabbit polyclonal to interferon-β (Biotin; ab84258) purchased from Abcam (Hong Kong, China). 

Briefly, wells of a Nunc-Immuno (MediSorp–VWR, Singapore) microtiter plate were coated with the capture antibody, wrapped with an adhesive plastic and incubated overnight at 4 °C. Coated plates were rinsed three times with washing solution (Tris-Buffered Saline/Tween), patted dry on a paper towel and blocked for 2 h. Blocking buffer was removed and plates were rinsed three times with washing solution. Samples were added to the plate and incubated overnight at room temperature, followed by washing (three times with washing solution). Detection antibody was then added, incubated for 2 h at room temperature and washed (three times with washing solution). Avidin-horse radish peroxidase (HRP) conjugate was added, incubated for another 1 h at room temperature and washed (three times with washing solution). Lastly, tetramethylbenzidine (TMB) reagent was added and incubated 5–30 min. To stop the reaction, 0.18 M H_2_SO_4_ was added to each well and absorbance at 450 nm was determined with a microplate reader.

### 4.6. Statistical Analysis

Results obtained from three independent repeats were presented as mean ± standard error of mean (SEM) for individual experiment. Comparisons between experiment and control were performed using Student’s *t*-test with GraphPad Prism (GraphPad, La Jolla, CA, USA). *p* < 0.05 was considered statistically significant.

## 5. Conclusions

While small molecule inhibitors targeting innate immune response are often applied in virology studies, their impact on mRNA transfection in human fibroblasts is relatively unknown. Results from our current study suggest that some small molecules, when applied in the context of mRNA delivery, are effective at inhibiting IFN production but also inhibited mRNA translation. We conclude that small molecules reported in this study, including some published IFN inhibitors, do not enhance in vitro mRNA transfection efficiency in human fibroblasts.

## Figures and Tables

**Table 1:** Small molecule inhibitors and respective mechanisms of action.

Group	Name	Mechanism	Cell Type	FDA Approval [19]	Reference
**Cardiac Glycosides**	Ouabain	Direct inhibition of sodium-potassium ATPase pump of RIG-I, indirect inhibition of IRF3 and NFĸB	HEK 293T	No	[20]
	Bufalin			No	
	Gitoxigenin			Yes	
	Digoxin		BxPC3 pancreatic cancer cells	No	[21]
	Proscillaridin		Glioblastoma cells (GBM6, GBM9, U87-MG, U251-MG)	No	[22]
**Natural Compounds**	Andrographolide	Inhibition of NFĸB	HEK 293	No	[23]
	Rosolic acid	Inhibition of STAT3 and NFĸB	Glioblastoma cells (U737, T98G)	No	[24]
	Tetrandrine	Direct inhibition of ERK1/2, JNK1/2; indirect inhibition of NFĸB, IĸB	Human mast cells (HMC-1)	No	[25]
	Parthenolide	Inhibits TRIF and MYD88 dependent pathways	RAW264.7 murine macrophages	No	[26]
**Pathway Inhibitors**	Sertraline	Inhibition of TLR3	HEK293	Yes	[15]
	Fluphenazine			Yes	
	Trifluoperazine			Yes	
	Amlodipine besylate			Yes	
	PKR inhibitor (C16)	Inhibition of PKR	Murine embryonic fibroblasts	No	[27]
	7-Desacetoxy-6,7-dehydrogedunin (7DG)	Inhibition of PKR	J774 murine macrophages	No	[17]

FDA: Food and Drug Administration.

## Supplementary Materials

The following are available online at www.mdpi.com/2218-273X/7/3/56/s1, Figure S1: Histograms of relative GFP expression of BJ fibroblasts transfected with GFP mRNA following small molecules treatments.
