# Supplementary material for: Suppression of mRNA Nanoparticle Transfection in Human Fibroblasts by Selected Interferon Inhibiting Small Molecule Compounds
Source: Biomolecules. 2017 Jul 31;7(3):56. doi: 10.3390/biom7030056 (PMC5618237; doi:10.3390/biom7030056)

Supplementary Figure1. Histograms of relative GFP expression of BJ fibroblasts transfected with GFP mRNA following small molecules treatments.

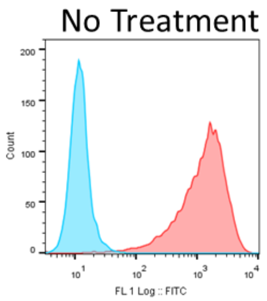

Cardiac Glycosides

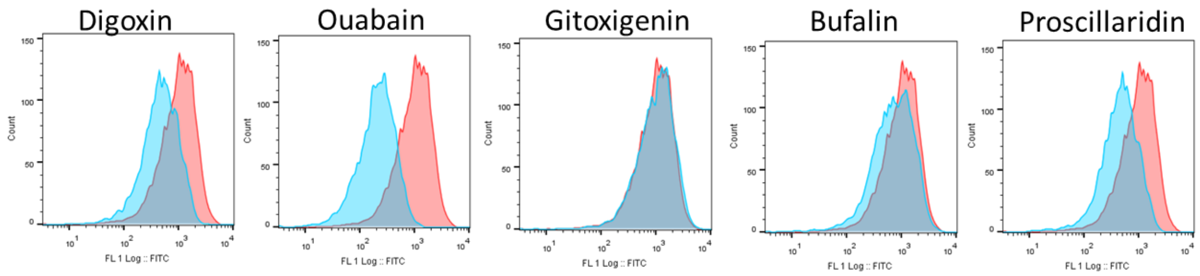

Natural Compounds

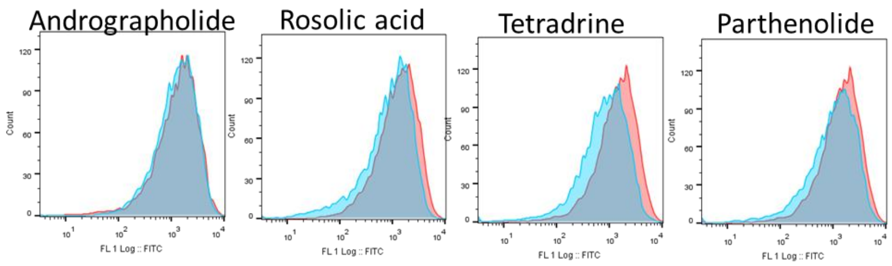

Pathway inhibitors

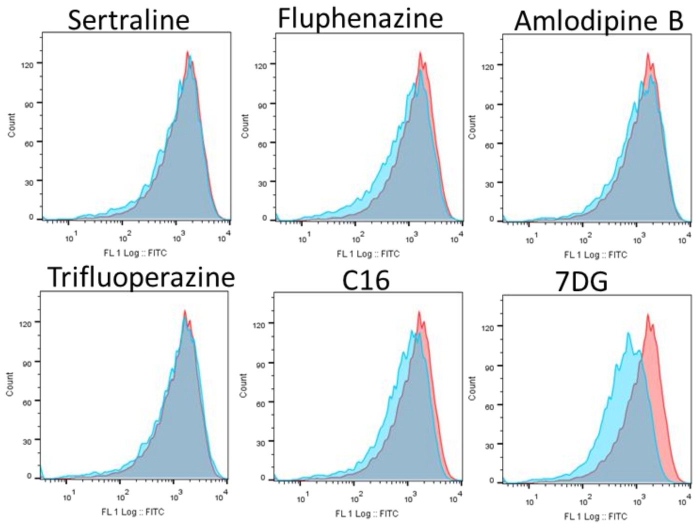

Supplement: Supplementary file 1 [file biomolecules-07-00056-s001.pdf]
